# Assessing the fluvial system resilience of the river Bacchiglione to point sources of pollution in Northeast Italy: a novel Water Resilience Index (WRI) approach

**DOI:** 10.1007/s11356-021-13157-5

**Published:** 2021-03-12

**Authors:** Domenica Mirauda, Donatella Caniani, Maria Teresa Colucci, Marco Ostoich

**Affiliations:** 1 School of Engineering, University of Basilicata, Viale dell’Ateneo Lucano 10, 85100 Potenza, Italy; 2 Provincial Department of Venice, Veneto Regional Environmental Prevention and Protection Agency (ARPAV), Via Lissa 6, 30172 Venice-, Mestre, Italy

**Keywords:** WRI, Water quality, River, Mathematical model, Point source pollution, Principal component analysis, Italy

## Abstract

Modelling and evaluating the resilience of environmental systems has recently raised significant interest among both practitioners and researchers. However, it has not yet been used to measure the absorption and recovery capacities of a river subject to varying levels of pollution due to natural and anthropic sources of contamination within the basin. Fast worldwide population growth and climate change are contributing to an increased degradation status in surface water bodies and to a decreased efficiency of their natural self-purification processes. Decision-makers are, therefore, more and more encouraged to implement alternative management strategies focussed on improving the system resilience to current and future perturbations. To this end, a novel Water Resilience Index (WRI), based on different quality parameters, was developed*,* and it is here proposed to estimate the ability of the river Bacchiglione, located in Northeast Italy, absorb continuous and unpredictable changes due to potential effects of point sources of pollution, that is*,* urban and industrial wastewater, and still maintain its vital functions. This new index is integrated in a mathematical model, which represents the river as an influence diagram where the nodes are the gauged stations and the arcs are the fluvial reaches among the stations, to identify the river reaches in need of resilience improvement. In addition, in order to simplify the analytical procedure and lower the costs and times of the monitoring activities, a *p*rincipal *c*omponent *a*nalysis is also used, as it is able to reduce the number of the water quality parameters to be collected from the sampling stations, distributed along the main river, and thus to calculate a minimum WRI. The good agreement between the results obtained by both the original and minimum WRI shows the effectiveness of the proposed methodology. This approach could be applied to all basins with the same issues, and not just in the Italian case study here analysed, as it might be a valid tool to plan interventions and mitigation actions, protecting the resource from pollution risks and achieving environmental quality and Sustainable Development Goals both in the water bodies and their surrounding territories. In addition, this strategy could be integrated in the existing models supporting local decision*-*makers and administrators, aiming at increasing the resilience of urban and rural areas to pollution phenomena and facilitating the development of effective policies to reduce the impacts of global change on water quality.

**Supplementary Information:**

The online version contains supplementary material available at 10.1007/s11356-021-13157-5.

## Introduction

Social and economic development, as well as the environmental sustainability of entire countries, increasingly depends on the quantity and quality of water supplies. Currently, approximately 2 billion people worldwide do not have access to reliable sources of drinking water. Moreover, freshwater is likely to become even less available in the future (by 2025 half of the world’s population will be living in water-stressed areas) due to both climate change and rapid population increase (WHO 2019). Clean and safe water determines the human and environmental survival so much that the detection of its contamination sources has been the main focus of the latest scientific research. Water quality degradation is caused by point and diffuse pollutants derived from natural processes (e.g. rainfall frequency and intensity, soil type and erosion, vegetation cover, river flow) and anthropic activities (e.g. agricultural, industrial and urban wastewater; malfunctioning and/or undersized treatment plants and distribution facilities). The improvement of surface water quality, deteriorated by the presence of excessive nutrients from highly populated areas (Viviano et al. 2014), is among the major challenges in Europe (Hering et al. 2010) and on a global scale (Smith and Schindler 2009). This issue will attract more and more attention, since the number of people in urban areas is increasing every year and it is likely to reach values of about 66% of the total global population by 2050 (Salerno et al. 2018; Viccaro and Caniani 2019). Therefore, it is very important to accurately monitor and correctly evaluate the impacts of urbanisation on the dynamics of water quality (Caniani et al. 2016). The problem is further exacerbated by the current climate change which, exposing rivers to frequent and extreme variations of temperature and fluvial regime (droughts and floods), contributes to the increase of their pollution and degradation state, to the efficiency decrease of natural purification processes, and to the rise of detrimental effects on aquatic life. Under the urbanisation and climate change effects, rivers are becoming increasingly vulnerable, and the existing risk management methods, dealing with known and quantifiable hazards, are no longer sufficient. These traditional risk-based strategies have been implemented to mitigate or avoid potential disruptive events and their impact (Kanakoudis 2004; Kanakoudis and Tolikas 2002, 2004; Blackmore and Plant 2008). However, recent experiences of natural and man-made water-related disasters in the world, and in particular in Italy, suggest that the water infrastructures as well as the current protection and prevention interventions may not be completely reliable, due to the high uncertainty and random failures resulting from unpredictable occurrences (Little 2002; Butler et al. 2014; Amarasinghe et al. 2016). In support of the conventional methods, river resilience management might help anticipating damages and reducing their impacts. In the last decades, first with the Hyogo Framework for Action of 2005 and then with the Sendai Framework for Disaster Risk Reduction 2015-2030, resilience measures have been given an important role in the decision-making process of developing strategies for readiness, response, and recovery of water infrastructures against unexpected, disruptive phenomena (Sharifi and Yamagata 2016; Shin et al. 2018). More recently, following the pandemic crisis caused by COVID-19, the European Commission has presented the targets of the recovery and resilience plans for the member states, which in the period 2021–2027 will start the Next Generation EU programme. These plans, and in particular the Italian one, have among their main goals that of strengthening the mitigation measures and the resilience of water infrastructures and bodies against extreme climate events. Resilience is widely interpreted as the capacity of a system to resist (preparation phase), absorb and withstand (responding phase), and rapidly recover from (restoration phase) exceptional conditions (Butler et al. 2016; Hosseini et al. 2016). Researchers have identified two types of resilience (Folke et al. 2002): the ecological one, which is the amount of disturbance that an ecosystem can absorb before it shifts into a new regime, controlled by a different set of ecological processes (Holling and Gunderson 2002); and the social one, which is the adaptation capacity of social systems to continuous changes (Adger 2000). However, a holistic approach that considers both the ecological and social resilience can be more effective in managing complex areas, which include an ecosystem and a social system together, and in planning appropriate mitigation measures as well as cost/benefit analysis of different actions. In other words, the quantitative measurement of resilience can provide various benefits such as understanding and comparing the system reaction under different environmental, social, and economic conditions; identifying vulnerable parts that need improvement with resilience strategies; and enhancing the planning of mitigation measures to reduce the risks of a bad management of the water resource. Unfortunately, despite these benefits, resilience measures and strategies have been rarely applied to the quality estimation of water bodies (Sarang et al. 2008; Hoque et al. 2012), and most studies have been focussed, instead, on the design of water quality indices (WQIs), able to describe the biological, chemical, and physical characteristics of freshwater for direct human consumption and other uses. In the last decades, such indices have been developed to monitor and evaluate the spatial and temporal changes of water quality and have been applied to several rivers (Pesce and Wunderlin 2000; Debels et al. 2005; Kannel et al. 2007; Sharma and Kansal 2011; KoÇer and Sevgili 2014; Sun et al. 2016; Zeinalzadeh and Rezaei 2017; Wu et al. 2018, Tripathi and Singal 2019; Jehan et al. 2020; Ustaoğlu et al. 2020; Njuguna et al. 2020; Wu et al. 2020; to name a few).

Most of the above-mentioned studies developed a WQI with fewer parameters by using various multivariate statistical techniques, such as cluster analysis (CA), principal component analysis (PCA), factor analysis (FA), and discriminate analysis (DA), in order to reduce the repetitive or correlated environmental variables and lower the analytical and monitoring cost.

The present paper, instead of focussing only on the sources of pollution as in the previous literature studies, analyses the resilience of the water body to the pressuring stresses through a novel Water Resilience Index (WRI), aimed at detecting the reaction of the river Bacchiglione to potential impacts of point source pollution (urban and industrial wastewater). In particular, the Bacchiglione basin belongs to the group of basins of Northeast Italy (Isonzo, Tagliamento, Livenza, Piave, Brenta, and Adige), whose achievement of the environmental quality objectives is governed by the fluvial management plan referred to the same Water District Authority according to Water Framework Directive (WFD) 2000/60/EC. These rivers are influenced by diffuse pollution from agriculture (fertilisers and pesticides) and by point pollution caused by discharges of wastewater plants (in some cases also non-treated wastewaters when the overflow systems are activated). In detail, if treatment of raw sewage is not sufficient, there are effects of oxygen consumption along the river, of nutrient (nitrogen and phosphorus) enrichment in sensitive areas (lakes, lagoons, etc.), with consequent eutrophication phenomena and negative impacts on the quality of bathing waters of the North Adriatic Sea (109 km of coast in the Veneto region), especially during intense precipitation events. Most of the times, these impacts influence the bathing activities during the spring-summer period, not matching the requirements of Directive 2006/7/EC 2006. The choice of the Bacchiglione basin is due to the deterioration of its water quality, caused by a great increase in population and industrial settlements, especially in its final breaches and near the big urban areas. In addition, the basin is influenced by a strong tourist presence and thus requires appropriate actions of safeguard and enhancement of its natural resources such as its water.

The WRI was calculated considering six water quality parameters, such as dissolved oxygen (DO), biological oxygen demand (BOD_5_), ammonium (NH_4_-N), nitrate (NO_3_-N), total phosphorus (TP), and *Escherichia coli* (*E. coli*), collected over the period 2008–2017 from twelve sampling stations distributed along the main reach of the river. Subsequently, a minimum WRI (WRI_min_) was implemented based on only two parameters selected through the PCA. This latter index does not allow having an exhaustive picture of the river quality status, but it can be useful to focus on some parameters more than others, especially when very low budget is allocated to monitoring activities. Therefore, the PCA technique was here used to eliminate the redundant data and to choose those parameters that mostly contribute to water pollution. Evaluating the ability of the whole water body to resist and to absorb the point sources of pollution of anthropic origin is a rather challenging objective and cannot be achieved by the resilience of each one of the components individually. Their connections and interactions with the surrounding environment must also be considered. Having said that, a global estimation of the fluvial system resilience cannot be performed without mathematical modelling, aimed at simulating a real trial based on numerous and complex correlations among different, dynamic entities, in both physical and functional terms. This implies a certain degree of abstraction in the reality under examination. It is necessary to identify the extent to which the aspects being considered impact the dynamics of the scenario under observation, by recognising the analytical functions which simulate the effects, albeit approximately. A methodological approach that may inspire the design and development of such a conceptual structure is to include both indices in a mathematical model, based on the theory of influence diagrams (Howard and Matheson 1984; Shachter 1988; Shenoy 1992). Specifically, the model outlined the river as an influence diagram where the nodes symbolise the cross sections, in which the gauged stations are located, while the arrows, representing the relationships between the functionalities of the different entities, depict the fluvial reaches linking the nodes. The influence diagram is a knowledge representation that can be viewed at three hierarchical levels: topological, functional, and numerical. In the present work, the influence diagram was considered only at a topological level, without involving the functional or numerical levels dealt with in previous studies (Howard and Matheson 1984; Shachter 1988; Shenoy 1992) and thus not needing a probabilistic basis. At the topological level, the nodes in the influence diagram represent the important variables in the system being modelled, and the direct links identify the conditional influences among them. In detail, a specific variable *x* is said to influence a variable *y* if the given information about *x* tells you something about *y*. This type of influence implies the existence, in the influence diagram, of the direct link (*x*, *y*). This application is widely present in literature and in particular in the study of social (Newman 2002; Newman et al. 2002), biological (Rubinov and Sporns 2010; Gao et al. 2016), business (Hearnshaw and Wilson 2013; Kim et al. 2015), communication (Albert et al. 2000; Doerr and Hernandez 2010), and transportation (Chopra et al. 2016; Zhang et al. 2015) networks, where the topological features reveal their strong impact on the resilience system (Meng et al. 2018). Limited topological studies exist on rivers, and the graph theory has mainly been applied to establish the vulnerability scenarios of highly anthropised fluvial reaches subject to flood and landslide events (Minciardi et al. 2006; Pascale et al. 2010; Sdao et al. 2011). Only recently the same authors (Mirauda and Ostoich 2011, 2018, 2020; Mirauda et al. 2018) have focussed their attention on the water quality but still from a vulnerability point of view. However, no comprehensive studies have been reported on the relationship between resilience and various topological characteristics of rivers, which is still unknown. Therefore, this knowledge gap needs to be addressed so that effective measures can be developed in the future to make fluvial reaches more resilient.

The aim of this paper is to propose a detailed and systematic assessment of the river capacity to react to point sources of pollution through the development of a novel WRI and by representing the river as an influence diagram. This approach considers the fluvial system as a bunch of elements (reaches and cross-sections) which, continuously interacting with one other and with the surrounding environment, influence the river response to the different contamination pressures in the basin. To this end, understanding of the environmental problem and detecting possible solutions are not based on a mere estimation and mapping of resilient fluvial areas but on the interrelationships among natural dynamic systems and physical, biological, social, and economic elements due to human activities. This way, the combined methodology might be useful in the future as a supporting tool for local administrators in the choice of interventions to increase the river resilience capacity in the shortest time possible but also to simplify and to smoothly communicate the complex information about the river quality status to the several stakeholders. In addition, the implementation of one WRI_min_ based on the acquisition of only two parameters facilitates a better management and prioritisation of the control programmes, increasing both the recording frequency of these parameters and the amount of measuring sites when the budget allocated to the monitoring activity is high, or otherwise focussing on the fluvial reaches at the highest risk of pollution when the same budget is very low.

The paper is organised as follows: in the ‘Case study’ section, the study area and data collection about water quality and quantity and sewage treatment plants are introduced; the ‘Methodology’ section describes the methodological approach based on the WRI and the mathematical model; in the ‘Results and discussion’ section, the discussion and the comparison of the resilience scenarios over the years and seasons, derived from the implementation of the original and minimum WRI in the model, are reported; finally, the ‘Conclusion’ section states the conclusions.

## Case study

### Basin characteristics

**Fig. 1:**
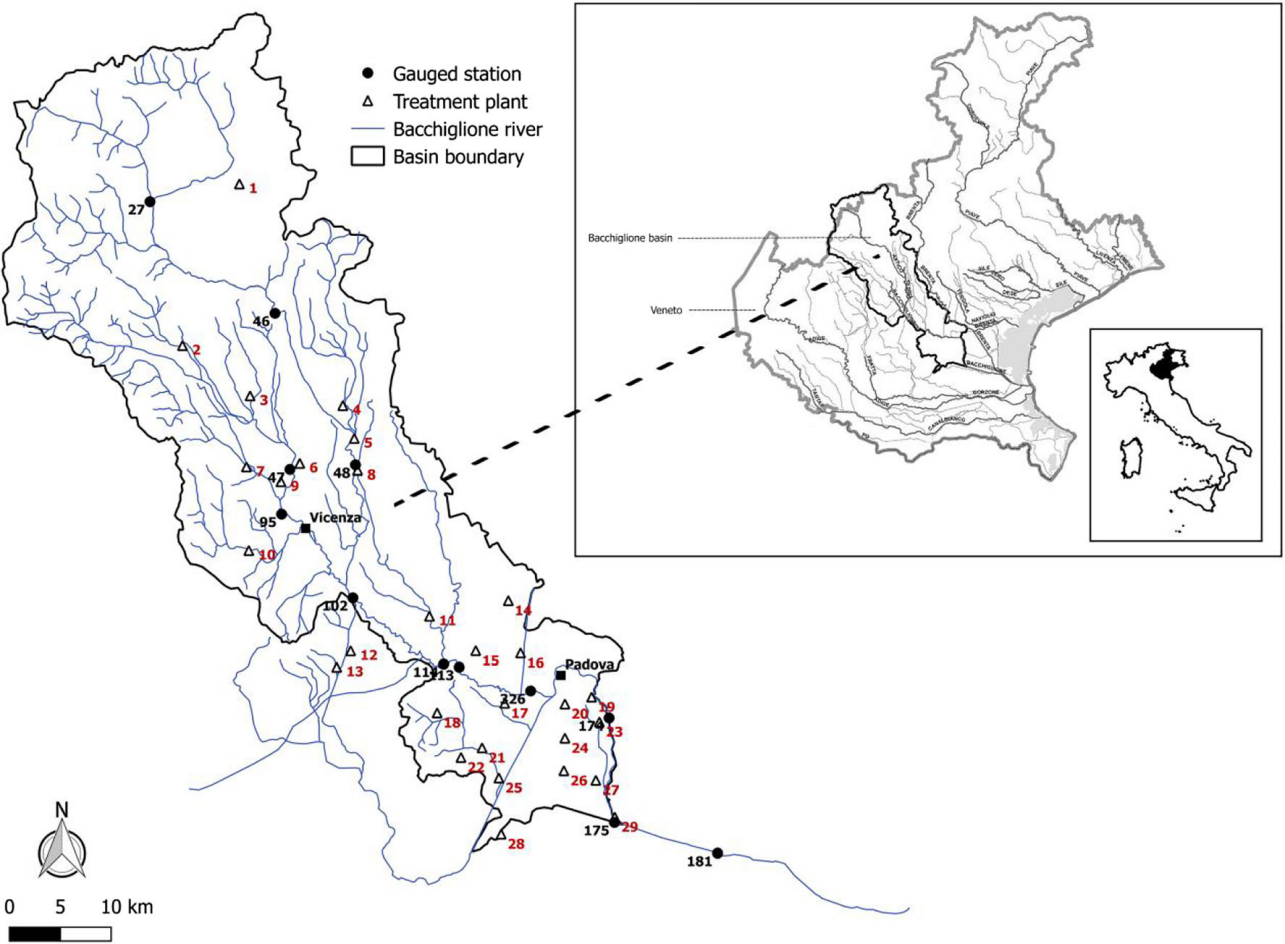
Bacchiglione basin with the gauged sections and treatment plants

The Bacchiglione basin is located in Northeast Italy and has an extension of 1177 km^2^ with a maximum elevation of 2334 m. It borders to the southwest with the basin of the Agno–Guà, to the west with that of the Adige, and to the Northeast with that of the Brenta (Fig. 1) (Mirauda and Ostoich 2011, 2018, 2020; Mirauda et al. 2018).

The river Bacchiglione has a length of 119 km; it originates from Dueville springs and crosses two major cities, Vicenza and Padua, flowing into the Adriatic Sea. The main stream is characterised by the presence of gravel boulders and cobbles, where the groundwater of Venetian Pre-Alps joins alluvial waterproof layers (Mirauda and Ostoich 2011, 2018, 2020; Mirauda et al. 2018).

The river undergoes seasonal variations of the flows: generally, the highest values of water discharge in winter, and the lowest ones in summer. The average annual precipitation is 1106 mm and rainfall mainly occurs from November to March (Mirauda and Ostoich 2011, 2018, 2020; Mirauda et al. 2018). The temperature increases from north to south with an annual mean value of about 14°C.

**Fig. 2:**
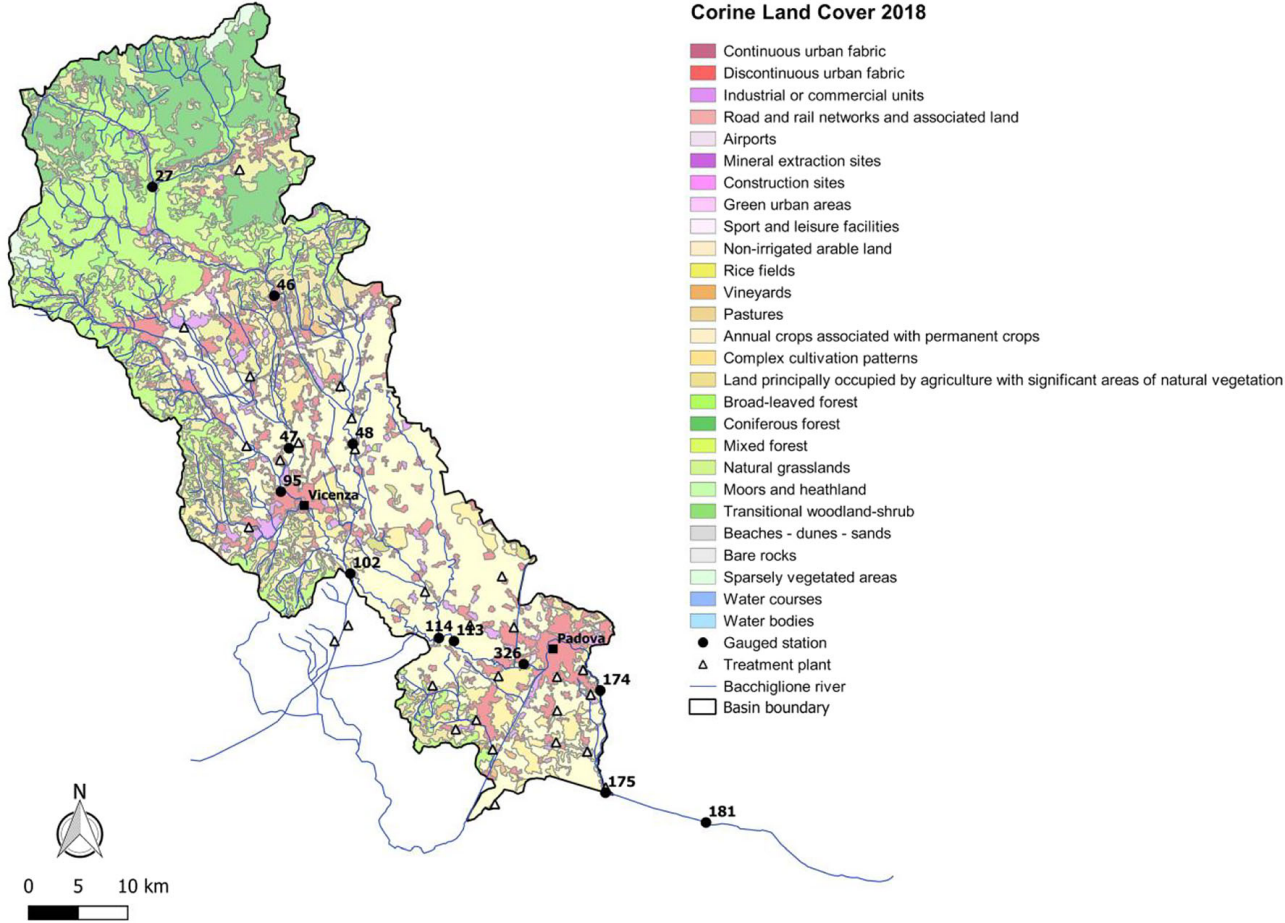
Land use in the Bacchiglione basin

As evidenced by mapping the Bacchiglione basin in Fig. 2, where only the land use typology with percentages over 0.5% are depicted, the mountain area is covered by broad-leaved and coniferous forests, which have decreased from 6 to 4% in the last years due to deforestation actions. The remaining area up to the mouth, including the cities of Vicenza and Padua, is mainly constituted of non-irrigated arable land, increased by about 28% since 2012, with small spots of discontinuous urban fabric. Besides, the industrial and commercial sites are concentrated near the two cities, while the Vicenza Pre-Alps area, occupied by the mineral extraction sites, is small (0.6%), with a slight decrease of 0.05% since 2006 (Mirauda and Ostoich 2011, 2018, 2020; Mirauda et al. 2018).

The basin primarily shows three distinct typologies of vegetation: aquatic plants (hydrophytes, floating buttercup, yellow and white lily pads, duckweed) within the river, cane fields along the banks, and shrubbery (nettle, medical plants) and trees (willows, alders, great ashes, elms) in the hinterland (Mirauda and Ostoich 2011, 2018, 2020; Mirauda et al. 2018). The fauna is especially linked to the flora, and the most common type is ornithofauna (native fauna), and the most easily observed aquatic species are moorhens and glens. The river Bacchiglione is the ideal environment also for fish such as salmonids, barb, zander, carp, and eel.

### Water quality data

The water quality data, provided by the Regional Environmental Prevention and Protection Agency of Veneto (ARPAV), were collected monthly from January 2008 to December 2017 at 12 monitoring sections located along the main reach of the river, nos. 27, 46, 47, 48, 95, 102, 113, 114, 326, 174, 175, and 181 (Fig. 1). Six water quality parameters, such as DO, BOD_5_, NH_4_-N, NO_3_-N, TP, and *E. coli*, measured according to the National Environmental Quality Standards for Surface Water (Legislative Decree No. 152/2006), were used to analyse water quality changes.

Previous studies of the authors (Mirauda and Ostoich 2011, 2018, 2020; Mirauda et al. 2018) showed how the major causes of pollution in the river Bacchiglione point contamination loads produced by the discharges of domestic and industrial wastewater, which have not been appropriately treated or eluded by the treatment plants. In detail, these works highlighted a better water quality in the mountain river reaches, due to the presence of forest areas where the level of disturbance from human activities is very low, and a deterioration of the water quality near the big urban areas and in the final part of the basin.

**Fig. 3:**
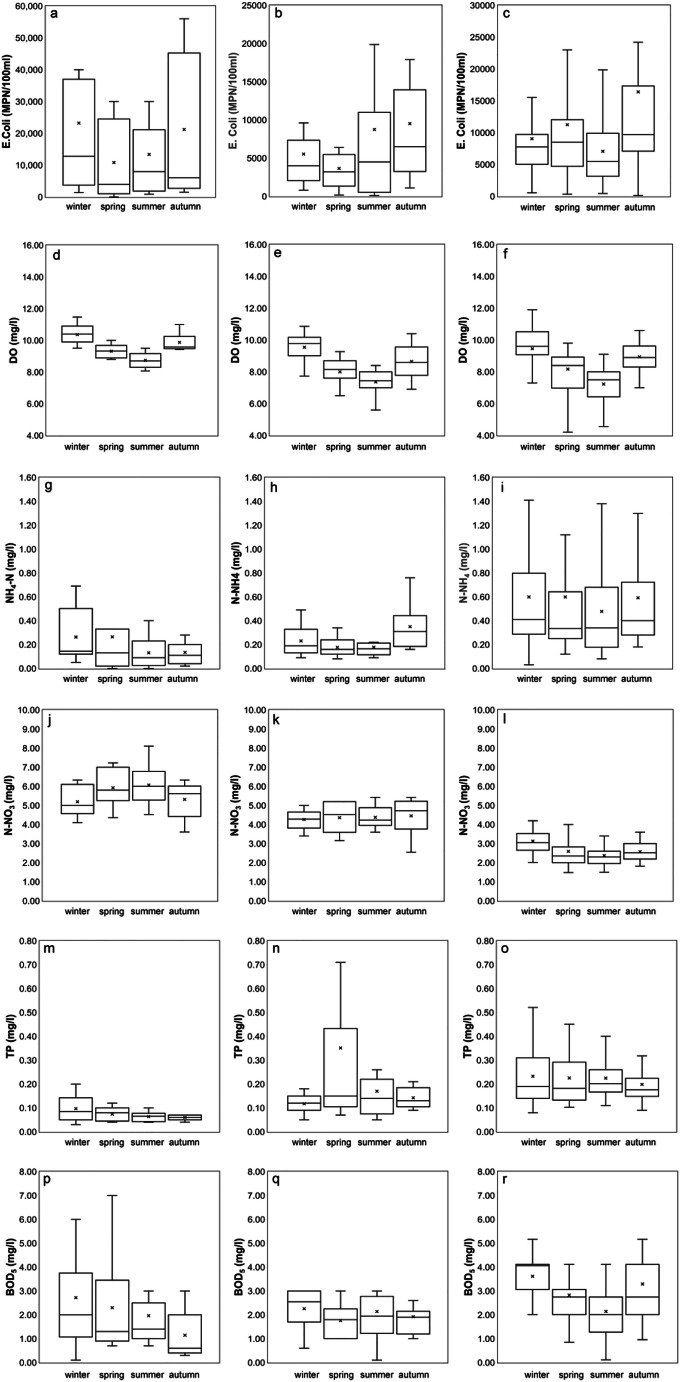
Seasonal trends of quality parameters at the gauged stations no. 95 (left, upstream of Vicenza), no. 102 (centre, downstream of Vicenza and upstream of Padua) and no. 174 (right, downstream of Padua)

Therefore, the analysis of water quality was mainly focused on three sample gauged stations located upstream and downstream of two cities and, in particular, no. 95 (upstream of Vicenza), no. 102 (downstream of Vicenza and upstream of Padua), and no. 174 (downstream of Padua). As shown in Fig. 3, the concentration of *E. coli* is bigger in the winter and autumn months with the highest recorded value at the station no. 95 in winter (23225 MPN/100 ml) and at the one no. 174 in autumn (16396 MPN/100 ml). The lowest values are, instead, 7073 MPN/100 ml in summer at the station no. 174 and 3648 MPN/100 ml in spring in the central part of the basin.

The DO concentration of the river Bacchiglione is quite high: in the summer months, the average DO values vary between 7.23 and 8.73 mg/l, while in the winter season they are between 9.45 and 10.36 mg/l (Fig. 3). The lowest DO values recorded in summer could also be linked to a greater activity of microorganisms, requiring oxygen for organic matter degradation (Yang et al. 2007). Spatially, the maximum concentrations of DO are recorded in the station no. 95, while lower values are observed at the stations near the cities, due to the discharge of domestic wastewater which reduces the quantity of oxygen.

The variation of nitrogen nutrients does not show significant seasonal changes (Fig. 3). In particular, for the ammonium, the winter/summer variation is around 0.10 mg/l for the station no. 95 and no. 174, while it is a lot lower for the station no. 102, around 0.05 mg/l. In the case of nitrate, instead, an increase of about 14% is observed in the summer at the station no. 95 and of about 2% at the central one, while for the station no. 174, there is a decrease of about 24%. Along the river, the behaviour of the two parameters is opposite, with an increasing trend towards the south for NH_4_-N with the highest values in winter (0.60 mg/l) and a decreasing trend towards north for NO_3_-N with the highest values in summer (6.07 mg/l).

Even the TP increases going downstream, as well as the ammonium. In fact, its highest values are recorded at the station no. 102 (close to the discharge points), with a mean concentration of 0.35 mg/l in spring, and at the station no. 174 in the winter periods, with a mean concentration around 0.23 mg/l (Fig. 3). The presence of nitrogenous and phosphorous compounds indicates a mixed origin due to a potential effect of leaching and runoff from fertilised farmland and effluent from certain industries (e.g. food processing) and domestic waste. However, these parameters do not exceed the national/international limit values and demonstrate how the basin is not subjected to pollution sources from agricultural activities.

Finally, the BOD_5_ parameter in the Bacchiglione basin shows values between 1.14 (no. 95 in autumn) and 3.53 mg/l (no. 174 in winter) with insignificant variation between seasons (Fig. 3). Overall, the values of BOD_5_ along the river slightly change when going from upstream to downstream, with higher values in the winter and spring months.

### Water quantity data

**Fig. 4:**
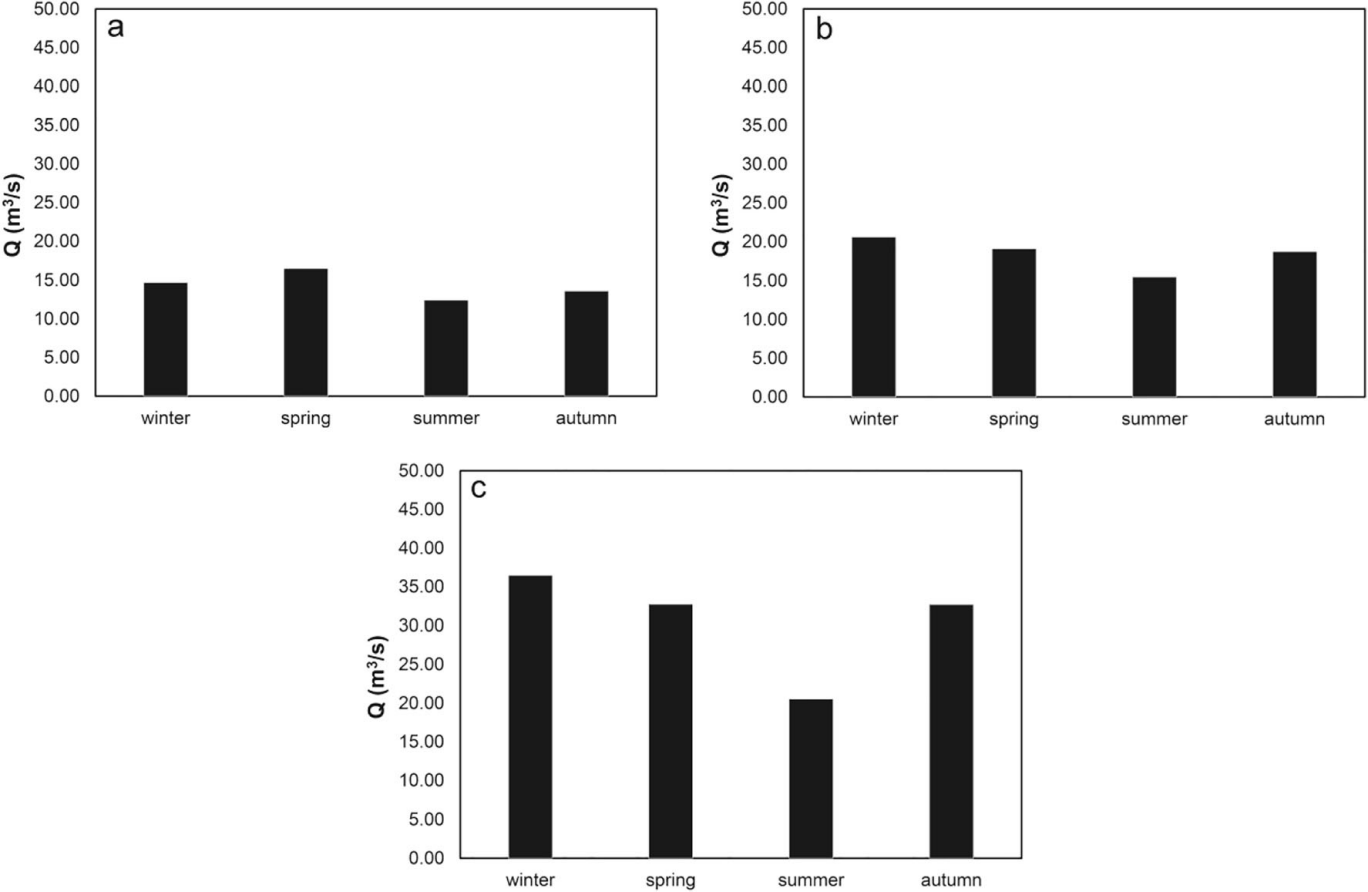
Seasonal trends of water discharge at the gauged station no. 95 (**a**), no. 102 (**b**), and no. 174 (**c**)

The water discharge values were determined through the direct measurement of the velocities and the area of the cross sections where the gauged stations are located or analytically obtained from theoretical stage-discharge relations. The point velocities were acquired both with classical current metres or sophisticated acoustic sensors and applying international standard methods and techniques derived from the ISO rules (ISO 748 1997; ISO 1100-2 1998). For the same reference stations used in the quality parameters analysis, that is, nos. 95, 102, and 174, the trend of the water discharges seasonally averaged from 2008 to 2017 is reported in Fig. 4.

At the gauged station no. 95, the water discharge trend shows a low variability all year round, and higher values are observed particularly in the winter and spring months. Continuing along the river, after the city of Vicenza, the average values of the flow rate increase of 29%, 14%, 20%, and 27 % in winter, spring, summer, and autumn, respectively, deriving from tributary branches and from the large presence of urban and industrial wastewaters. Finally, in the terminal part of the river Bacchiglione, downstream the city of Padua, a significant increase is recorded with doubled values in almost all months except for the summer ones (44% in winter, 42% in spring, 25% in summer, and 43% in autumn), due to the great increase of urban wastewater discharge.

### Sewage treatment plants

For the design of the resilience index WRI, only the treatment plants with a potential of more than 2000 equivalent inhabitants (EI) were considered, because they release more pollution loads and flow rate (Fig. 1). Thirty-two percent of the investigated treatment plants are planned with primary and secondary (activated sludge aerobic reactor) sections. These plants can guarantee an abatement efficiency of 60–95% of organic load, 30–40% of nutrient load (Metcalf and Eddy 2003), and at least two logs of 50–60% of microbiological load (Ragazzo et al. 2007). The remaining 68%, having a potential of more than 10000 EI, is also equipped with a tertiary treatment section. These plants are able to abate not only organic pollution but also nitrogen and phosphorus compounds, even though they cannot reduce substances that are toxic for microorganisms. Therefore, they reduce only the loads responsible for (1) oxygen exhaustion in the river due to organic load and to the resulting biological activity of heterotrophic microorganisms; (2) algal blooms in reservoirs, lakes, estuarine zones, lagoons, and coastal zones, due to nutrient enrichment phenomena; and (3) dangerous effects of pathogens caused by the domestic use of water. The abatement of almost all the microbiological loads is guaranteed only through a disinfection section (Zann and Sutton 1995). However, the microbiological load does not appear so important unless related to human use (e.g., drinking water production, irrigation, bathing) and can produce dangerous by-products (Ostoich et al. 2013) with toxic effects on the aquatic ecosystem (Ostoich et al. 2010) when the disinfection is done with different technological solutions (chlorine compounds, peracetic acid, performic acid, ozone, UV rays, ultra-filtration, etc.). Table 1 reports the number of EI and type of treatment for each plant located within the basin.

**Table 1:** Equivalent inhabitants and type of treatment for each plant within the Bacchiglione basin

Code	City	EI	Type of treatment
1	Vicenza	20,000	Tertiary
2	Vicenza	60,000	Tertiary
3	Vicenza	127,000	Tertiary
4	Vicenza	9000	Primary and secondary
5	Vicenza	3000	Primary and secondary
6	Vicenza	22,000	Tertiary
7	Vicenza	40,288	Tertiary
8	Vicenza	3000	Primary and secondary
9	Vicenza	13,500	Tertiary
10	Vicenza	12,500	Tertiary
11	Vicenza	28,500	Tertiary
12	Vicenza	5500	Primary and secondary
13	Vicenza	2000	Primary and secondary
14	Padua	12,000	Tertiary
15	Padua	12,800	Tertiary
16	Padua	22,000	Tertiary
17	Padua	20,000	Tertiary
18	Padua	7000	Primary and secondary
19	Padua	197,000	Tertiary
20	Padua	13,000	Tertiary
21	Padua	35,000	Tertiary
22	Padua	5000	Primary and secondary
23	Padua	18,000	Tertiary
24	Padua	40,000	Tertiary
25	Padua	20,000	Tertiary
26	Padua	5933	Primary and secondary
27	Padua	5000	Primary and secondary
28	Padua	7500	Primary and secondary
29	Padua	2000	Primary and secondary

## Methodology

### Water Resilience Index

The load of pollutants acting on the basin depends on numerous factors, such as the distance from the pollution source, the river water discharge influencing the dilution processes, the natural self-depuration, and the environmental conditions (e.g. temperature), which can either accelerate or slow down the biodegradation phenomena.

In this paper, a new index, Water Resilience Index (WRI), is developed to measure the self-depuration capacity of a river from contamination point loads due to the malfunctioning and/or undersized treatment plants as well as untreated urban and industrial wastewater. It is defined as:

1 $$ {WRI}_i=\frac{c_i}{\sum \limits_{r=1}^n\frac{Q_{tr.p.,r}\cdotp {c}_{tr.p.,r}}{Q_{tr.p.,r}}} $$

where *c*_*i*_ is the concentration of the six water quality parameters (DO, BOD_5_, NH_4_-N, NO_3_-N, TP, and *E. coli*) measured by the gauged station, *i*, while *Q*_tr.p.,r_ and *c*_tr.p.,r_ are, respectively, the flow rate and the concentration of the six parameters discharged by the *n* treatment plants located upstream of the station, *i*. All the concentrations were normalised assigning a value between 0 and 100 in order to be independent on the dimensionality of the original data set and weighted using a scale, *Pi*, from 1 to 4, with 4 when the parameter has the most importance for aquatic life preservation, while 1 when this parameter has a smaller impact (Table 2). Low index value means that the river is less resilient to anthropic pressure sources deriving from urban wastewater discharges, while its high value indicates a dilution effect as well as a self-depuration capacity provided by the river itself. As already described in the introduction, the index was primarily implemented considering all the six quality parameters monitored along the main reach of the river, in order to have a complete picture of the river quality status. However, the elevated analytical costs and the scarce budget for environmental monitoring, sometimes, pushed this research towards the implementation of a WRI_min_ based on only two parameters, which were selected through the PCA described in Appendix. The results of the PCA suggest that the *E. coli* and DO, explaining the highest total variance in the whole dataset, could play a key role in affecting water quality changes and in identifying the impacts of anthropogenic activities and urban-pollution risk sources of the river Bacchiglione. Therefore, both parameters can be selected to calculate the WRI_min_. In particular, *E. coli*, which includes biological and nutrient parameters, may be mainly associated to point sources of municipal and industrial pollution, while DO can be indicative of the aquatic organisms growth and of the fluvial biodiversity conservation within the river. This latter parameter, being easily determined in the field, was also used by many researchers to build various minimum WQIs in different water bodies (Kannel et al. 2007; Pesce and Wunderlin 2000; Sanchez et al. 2006).

**Table 2:** Relative weight, *Pi*, and normalised values of water quality parameters (Kannel et al. 2007; Kaswanto et al. 2012)

Parameter	Pi	Normalisation factor										
		100	90	80	70	60	50	40	30	20	10	0
DO	4	≥7.5	>7	>6.5	>6	>5	>4	>3.5	>3	>2	≥1	<1
BOD_5_	3	<0.5	<2	<3	<4	<5	<6	<8	<10	<12	≤15	>15
NH_4_-N	3	<0.01	<0.05	<0.1	<0.2	<0.3	<0.4	<0.5	<0.75	<1	≤1.25	>1.25
NO_3_-N	2	<0.005	<0.008	<0.01	<0.04	<0.075	<0.1	0.15	<0.2	<0.25	≤0.5	<0.5
TP	1	<0.2	<1.6	<3.2	<6.4	<9.6	<16	<32	<64	<96	<160	>160
*E. coli*	3	<50	<500	<1000	<2000	<3000	<4000	<5000	<7000	<10000	≤14000	>14000

### Mathematical model

The main target of the present paper is not only the evaluation of the water body resistance to and absorption of anthropic point sources of pollution but also the definition of a methodological approach able to support urban planners and stakeholders during the land-planning phase, as it can be used to establish priorities of intervention and of resources allocation. To this end, a mere estimation and mapping of resilient fluvial areas is not the only concern, as the connection among the different fluvial reaches and their interaction with the surrounding environment can also influence the river response to pollution sources. In fact, a single reach should generally not be considered as an isolated unity, since it is functionally related to the other reaches belonging to the same fluvial system.

On these bases, the investigated river can be described as an influence diagram where the nodes are the cross-sections at the gauged stations and the arcs are the fluvial reaches between the stations.

Each node (entity) is subjected to an identified and quantified stress defined by point direct and indirect pressure from the surrounding territorial system. A stress vector, ${\overline{\xi}}_i^k$, referred to a specific hazard, k, is associated to each node, *i*, and is related to the intrinsic level of integrity of the node, *y*_i_*,* expressed through the resilience function, ${f}_i^k$:

2 $$ {y}_i={f}_i^k\cdotp \left({\overline{\xi}}_i^k\right) $$

From Eq. (2) the level of physical integrity is obtained. It corresponds to the resilience of the *i*-th node, which can depend on how the node adapts to a given stress caused by a natural hazard, *k*. Being the stress, ${\overline{\xi}}_i^k$, a scalar quantity, it can be identified by a specific index, *I*_i_, and Eq. (2) is a non*-*increasing monotonic function included in the co*-*domain [0, 1]:

3 $$ {y}_i=a\left(\frac{e^{-\alpha {I}_i^2}}{1+{e}^{-\alpha {I}_i^2}}\right) $$

where the parameters a and α define the shape of the function. In the present work Eq. (3) becomes:

4 $$ {y}_i=a\left(\frac{e^{-\alpha {WRI}_i^2}}{1+{e}^{-\alpha {WRI}_i^2}}\right) $$

However, the resilience of a territorial entity also depends on the functionalities of other elements, that is the intrinsic levels of functionality of the other nodes, ${\overline{x}}_j$, related to the *i*-th node:

5 $$ {\overline{x}}_{\mathrm{i}}={\overline{\upvarphi}}_{\mathrm{i}}\left({y}_{\mathrm{i}},{\overline{x}}_{\mathrm{j}},\mathrm{j}\upepsilon P(i)\right) $$

where *P*(*i*) is the set of predecessors, j, of node i, for which the oriented link (*j*,*i*) exists. In Eq. (5) the term ${\overline{x}}_j$ can be defined through a scalar monotonic non*-*decreasing function, with domain and co*-*domain belonging to the range [0, 1] (Balbis et al. 2002):

6 $$ {w}_{\mathrm{ij}}\left({x}_{\mathrm{j}}\right)=\left(1-0.1{\alpha}_{\mathrm{ij}}\right)\frac{\left(1-{e}^{-{\alpha}_{\mathrm{ij}}{x}_{\mathrm{j}}^2}\right)}{\left(1-{e}^{-{\alpha}_{\mathrm{ij}}}\right)}+0.1{\alpha}_{\mathrm{ij}} $$

where α_ij_ is a parameter characterising the link (*i*,*j*) and it has been computed considering the existent correlations between the entities *i* and *j*.

The resilience of each node described by the functional integrity, ${\overline{x}}_i$, varies from 0 (non-functional entity) to 1 (complete entity functionality). In the analysed case of the resilience phenomenon, the vector function, ${\overline{\varphi}}_i$, of the Eq. (5) is reasonably optimised by considering the maximum resilience for the river and the maximum system functionality (conservative condition). Equation (5) can be modified as:

7 $$ {x}_i=\mathit{\max}\left({y}_i,{w}_{ij}\left({x}_j\right),j:{e}_j\epsilon P(i)\right) $$

The following table summarises the main symbols used in the model (Table 3).

**Table 3:** List of main symbols

${\overline{\xi}}_i^k$	Vector stress referred to natural hazard of the *i*-th node
*K*	Natural hazard
${f}_i^k$	Resilience function of the *i*-th node
*y*_*i*_	Level of physical integrity of the *i*-th node
*a, α*	Coefficients of the level of physical integrity
${\overline{x}}_j$	Vector level of functionality of the *j*-th node linked to the *i*-th node
*w*_*ij*_*(x*_*j*_*)*	Scalar level of functionality of the *j*-th node linked to the *i*-th node
*α*_*ij*_	Coefficient of the level of functionality
${\overline{x}}_i$	Vector functional integrity of the *i*-th node
*x*_*i*_	Scalar functional integrity of the *i*-th node
${\overline{\varphi}}_i$	Vector function of optimisation of the *i*-th node
*P(i)*	Set of predecessors of the *i*-th node

The evaluation of the river resilience in the present paper required the calculation of the physical integrity of the single node, *y*_*i*_, and of the functionalities of other graph elements, *w*_*ij*_*(x*_*j*_*)*.

To calculate *y*_*i*_ by Eq. (4), the parameters *a* and *α* were determined formulating the hypothesis that, for limit values of the index (0,1), three resilience classes (0–0.4, 0.4–0.6, 0.6–1) have to be considered (low, mean, high). The function *w*_*ij*_*(x*_*j*_*)* was obtained by Eq. (6) on the basis of different correlations between the indices of the nodes. In particular, by assigning a value to each level of influence, it was possible to estimate the corresponding parameters *α*_*ij*_ (Table 4).

**Table 4:** Values associated to the correlations

Correlation *i*–*j*	Level of influence	*α*_*ij*_
Weak	0–0.3	8
Mean	0.3–0.6	6
Strong	0.6–1	2

It is important to underline that a gauged station located downstream will be influenced by one upstream of the same reach, but the opposite is not possible. The resilience of each single node, representing the high capacity of the corresponding fluvial reach to resist, absorb, and recover from potential point source contamination of the surrounding environment, was evaluated first obtaining the WRI with all of the six parameters and then the WRI_min_ considering only the two parameters selected through the PCA.

**Fig. 5:**
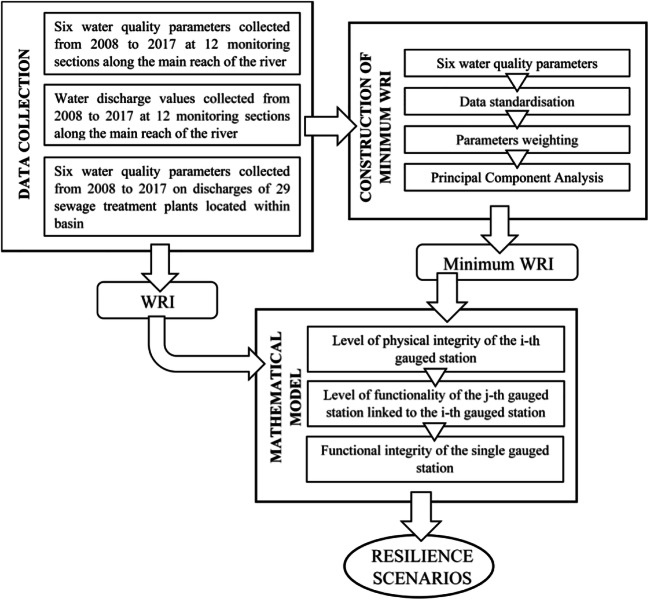
Flow chart summarising all the steps of the applied methodology

In Fig. 5, all the steps of the applied methodology described above are reported.

## Results and discussion

The obtained values of the WRI show high variability, from a minimum value of 0.514 to a maximum one of 1.984. The WRI is characterised by a mean of 1.227 and a standard deviation of 0.393. The Pearson’s correlation coefficients between the WRI and all the water quality parameters in all the gauged stations show a negative relationship everywhere except for DO (Table 5), which indicates that the WRI gives a good representation of the fluvial capacity to react to organic and nutrient pollutant effects in rural and urban landscapes. This underlines that the behaviour of the organisms inside the river system favours a status of equilibrium.

**Table 5:** Pearson’s correlation coefficients between WRI and water quality parameters

Water quality parameters	Pearson’s correlation coefficients
*E. coli*	−0.362
NH_4_-N	−0.617
NO_3_-N	0.258
TP	−0.795
BOD_5_	−0.448
DO	0.393

**Fig. 6:**
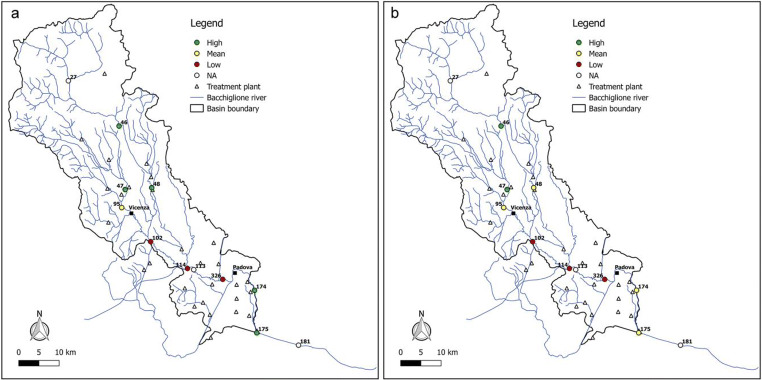
Resilience scenarios evaluated in 2008 with **a** WRI and **b** WRI_min_

**Fig. 7:**
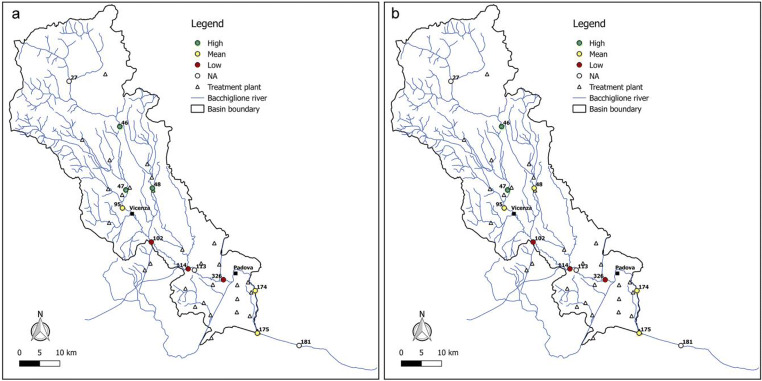
Resilience scenarios evaluated in 2010 with **a** WRI and **b** WRI_min_

**Fig. 8:**
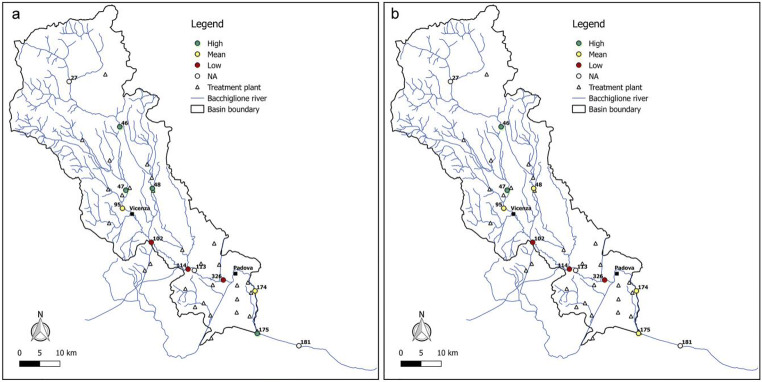
Resilience scenarios evaluated in 2012 with **a** WRI and **b** WRI_min_

**Fig. 9:**
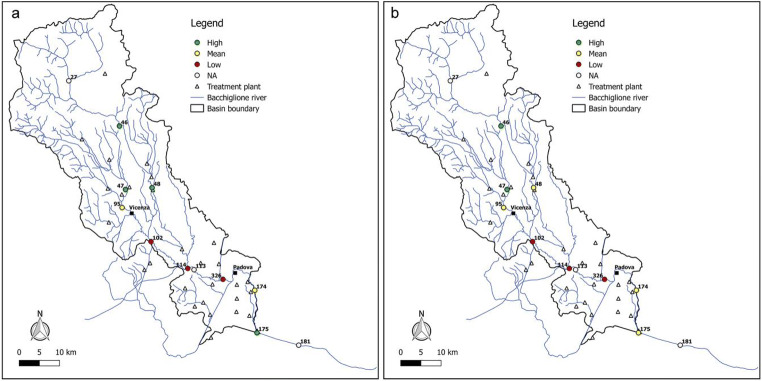
Resilience scenarios evaluated in 2014 with **a** WRI and **b** WRI_min_

**Fig. 10:**
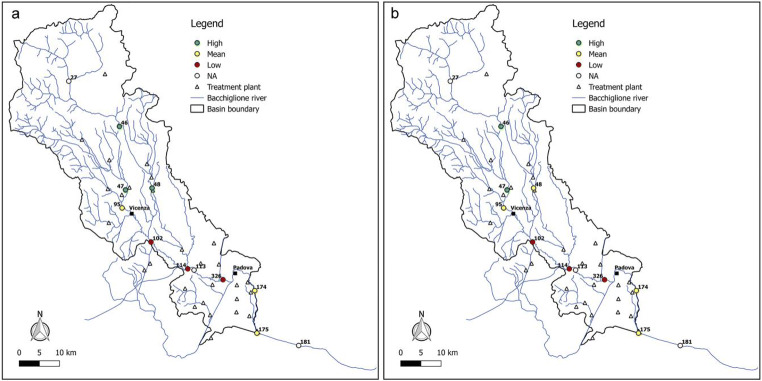
Resilience scenarios evaluated in 2017 with **a** WRI and **b** WRI_min_

Figures 6, 7, 8, 9, and 10 report for the years 2008, 2010, 2012, 2014, and 2017 the resilience scenarios evaluated considering both the original and WRI_min_. For nodes 27, 113, and 181, instead, the WRI and thus the resilience function were not calculated due to the absence of treatment plants upstream of the gauged station. From the figures, it is possible to note that the river Bacchiglione could be divided in three main clusters, according to their similar reactions to the impacts of external stresses: the area located upstream of Vicenza, the zone between the two cities, and the remaining part of the basin downstream of Padua.

The first area generally shows high resilience scenarios throughout the reference period 2008–2017. Such scenarios highlight a good self-purification capacity of the river, thanks also to continuous turbulence processes due to steep reaches in the upper part of the basin and to the presence of naturalised fluvial stretches, covered by broad-leaved and coniferous forests (Fig. 2). However, as demonstrated by the mean values of resilience in node 95 located immediately upstream of Vicenza, the river is not completely able to absorb the whole pollution load produced both by the washout of arable lands and by the discharging of insufficiently treated wastewater, deriving from small municipalities spread over the mountain area. This result confirms the high value of *E. coli* (Fig. 3) recorded in the station no. 95, especially in winter, and that of nitrate, due to agricultural runoff from fields treated with fertilisers and pesticides.

In order to improve the water quality in this fluvial reach, the local administrators should plan a series of long-term actions. They should be addressed, on one hand, to the adoption of best land use practices with the introduction of new cultivation and ploughing techniques in order to minimise the effects of chemical fertilisers, insecticides, and pesticides and, on the other hand, to the implementation of new treatment technologies in the existing plants.

The area between Vicenza and Padua shows, instead, a change in the resilience from medium to low over the years, underlining the greater inability of the river to absorb the significant pollution load. The homogeneity of land use and cover gives strength to the idea that the factors causing the degradation of the water quality are mainly related to the presence of urban and industrial areas, which have greatly increased over the last years. This underlines how the discharge of domestic and industrial wastewater contributes to the increasing of the contamination status highlighted by the presence of *E. coli*, ammonium, total phosphorus, and BOD_5_. Considering the level of contamination, the municipal decision-makers should intervene promptly with short-term actions to reduce the point pressure sources deriving from surrounding areas and increase the resilience of the river. For example, mitigation measures aimed at the re-naturalisation of some fluvial reaches with parks and green areas could be used to create artificial aeration facilities to improve the DO level and self-purification of the river. In addition, afforestation along the riverbanks would help to control siltation, erosion, agricultural runoffs containing pesticides and fertilisers, etc. Even the revamping of wastewater treatment plants with advanced technologies and the building of new treatment plants could be useful interventions to resolve the problem.

The last area downstream of Padua mainly depicts a mean resilience scenario throughout the reference period 2008–2017. This underlines the fact that the urban and industrial loads do not represent a significant threat for the river. In fact, a water quality higher than that of the previous reach demonstrates not only a greater resilience of the river to the pollution phenomenon but especially the positive effects deriving from the building of new treatment plants, following the Directive 2000/60/CE of 23/10/2000 2000 adoption. However, the presence of a mean resilience scenario underlines the influence of upstream reaches which are strongly polluted. In order to avoid fluvial reaches with a high level of contamination in the future, monitoring activities could be increased.

**Fig. 11:**
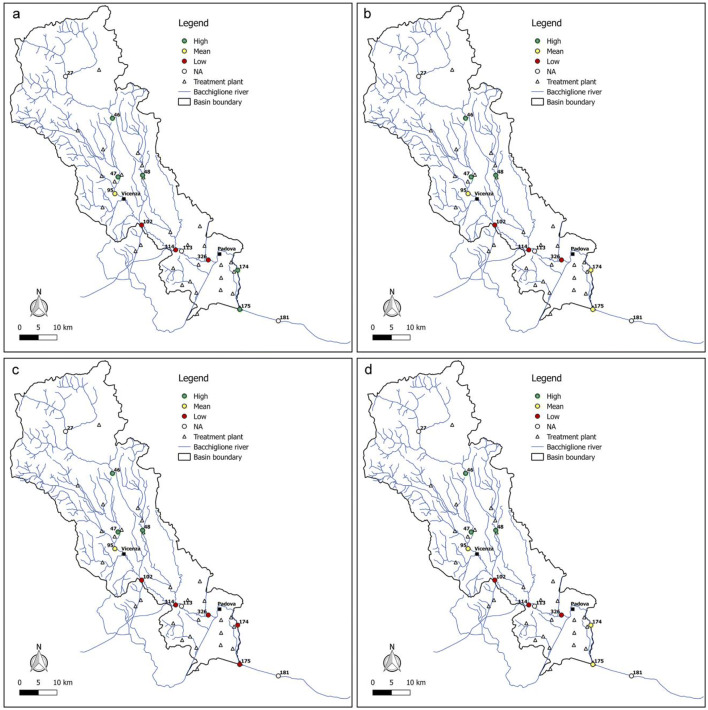
Resilience scenarios evaluated in 2017 with WRI in **a** winter, **b** spring, **c** summer, and **d** autumn

**Fig. 12:**
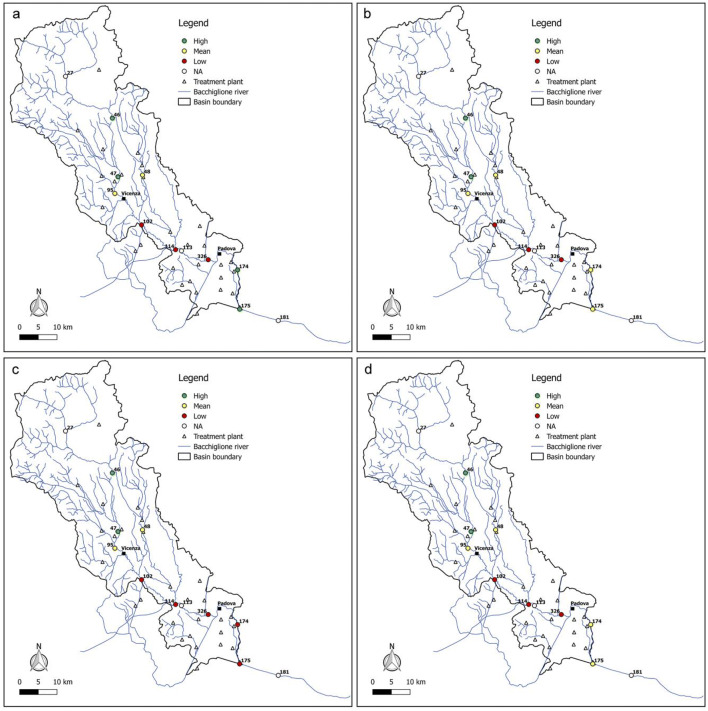
Resilience scenarios evaluated in 2017 with WRI_min_ in **a** winter, **b** spring, **c** summer, and **d** autumn

In addition, in order to know the seasonal river reaction to the pressures exerted by the pollution sources, the values for both indices in the sample year 2017 were reported (Figs. 11 and 12). As shown in the figures, upstream and downstream Vicenza city, the resilience of each reach is the same with varying season, while in the final part of the basin, the quality degrades during the summer and autumn months. This might be due to the fact that the lower runoff and flow velocity of the river during the dry season is not conducive to the dilution and diffusion of pollutants coming from the civil and industrial discharge of the cities, making the water quality worse in summer. In addition, the activity of microorganisms is suppressed in summer, resulting in a low self-purification ability of the river.

Overall, from all the figures, it is possible to observe that the trend of the resilience scenarios evaluated through WRI_min_ is similar to that predicted by the original index. In detail, in the first area upstream of Vicenza, both the original and the minimum WRI highlight the high capacity of the water body to resist to and absorb the point sources of pollution. This demonstrates that the *E. coli* and DO parameters have higher weight than the other variables and play a key role in the general resilience assessment of the river Bacchiglione, although they cannot provide a complete picture of the river quality status. This result is also reinforced by the PCA, which displays the positive correlations between *E. coli* and the other parameters (see Appendix). Even for the second cluster located between the cities of Vicenza and Padua, a perfect analogy between the indices is evident. A slightly different result is obtained, instead, in the final part of the basin, where in some years a medium resilience is recorded for the WRI_min_ compared to a high value observed for the original index. However, the worst scenario is the one shown by the minimum index, and thus, the chosen methodological approach seems to be strongly precautionary. The seasonal trend highlights that the season in which very low resilience is observed is the summer, when the water discharge and the flow velocity are lower and the dissolved oxygen quantity is the smallest, due to the high temperature.

The integrated use of WRI_min_ and the mathematical model allows a rapid and easy identification of the main contamination sources within the basin and of the fluvial reaches more subject to pollution risk. Furthermore, the acquisition of only two parameters facilitates a better management and prioritisation of the local monitoring programmes, increasing both the recording frequency of these parameters and the amount of measuring sites, especially in fluvial reaches at higher risk of contamination and located in strongly anthropised, industrial, and agricultural areas. In addition, the present methodology could be applied when the budget allocated to the monitoring activity is very low, demanding low time and cost of in situ surveys. Finally, the fast analytical evaluation suggests its use for an on-line water quality control.

## Conclusions

The present paper proposed a new Water Resilience Index (WRI) able to evaluate the ability of a river to react especially to contamination point sources of anthropic origin. This index was applied to a sample basin in Northeast Italy mainly stressed by high population density and numerous industrial settlements, which have increasingly grown over the years. In addition, a strong tourist presence all year round requires appropriate and continuous actions of safeguard and enhancement of the water resource.

A PCA through which some redundant variables were deleted was applied in order to identify the most responsible parameters in the water quality and reduce the costs and times of monitoring and analysis activities. This statistical technique is allowed obtaining an alternative WRI based on only two parameters, *Escherichia coli* and dissolved oxygen. The original and minimum indices were integrated in a mathematical model based on the graph theory in order to identify the river reaches in need of resilience improvement. This way, the river was considered as an ecosystem interacting with its territorial context and not as a separate element. The model results underlined a significant deterioration of the water quality from upstream to downstream during a 10-year monitoring period, caused by high population density, the presence of tourists, and numerous industrial settlements. The comparison between the two indices did not show substantial differences in terms of resilience scenarios.

The combination of the new WRIs and the mathematical model, based on the topological characteristics of the river, could be an efficient methodology to rapidly and easily identify the fluvial reaches able to resist, absorb, and recover from potential contamination point sources in strongly anthropised, industrial, and agricultural areas. In particular, this approach could be useful for local administrators and decision-makers to understand where to intensify monitoring activities and what mitigation measures and actions to implement in order to improve the resilience of the river. This way, the achievement of the Sustainable Development Goals (SDGs) could be accelerated, and in particular, an increased reduction of pollutants especially in fluvial environments (goals 6.3 and 14.1); the minimum release of hazardous substances and of untreated wastewater in rivers (goal 6.3); and the right use of the water resource (goal 12.2). This process could further be facilitated by the implementation of some mitigation measures and interventions such as the adoption of best land use practices and sustainable food production systems (goal 2.4), the re-naturalisation of some fluvial reaches with parks and green areas (goal 6.6), the revamping of wastewater treatment plants with advanced technologies (goal 6.A), and the building of new treatment plants (goal 6.A). In addition, the proposed combined approach could help to draw greater attention towards the employment of more and more efficient decision support systems (DSSs) and launch the implementation of technical guidelines informing the water authorities and managers on the pollution risks, especially in areas with high risk of growing population and climate change, on how the water body resists to external pressures, and on how to act in conditions of real or potential damage. This approach could be applied to all basins with the same issues, and not just in the Italian case study here analysed, in order to lead the local policies towards a more appropriate choice of development strategies and plans to increase the resilience of entire urban and rural territories.

Finally, if this methodology is implemented in an online platform, it could even help the fast and simple communication of the river quality status to the population, showing timely and continuous up-to-date information on various resilience scenarios, and it could facilitate communication among local administrators and stakeholders.

## Supplementary Information

ESM 1 (DOCX 710 kb)
