# Supplementary material for: Assessing the fluvial system resilience of the river Bacchiglione to point sources of pollution in Northeast Italy: a novel Water Resilience Index (WRI) approach
Source: Environ Sci Pollut Res Int. 2021 Mar 12;28(27):36775–92. doi: 10.1007/s11356-021-13157-5 (PMC7954523; doi:10.1007/s11356-021-13157-5)
Supplement: Supplementary file 1 — (DOCX 710 kb) [file 11356_2021_13157_MOESM1_ESM.docx]

**Appendix**

*Parameter selection through PCA*

The two parameters needed to obtain the WRI_min_ were detected through the PCA. For this purpose, all the parameters have been normalised through the software Matlab 2016a by calculating their z-score values, which have zero mean and unit variance. Z-scale standardisation was used in order to minimise the influence of the difference on the variance of variables and to adjust for the disparity in the variable sizes and in the measurement units. Subsequently, the PCA was applied both on all data and grouping them in the four seasons using the Varimax rotation to maximise the variance of the squared loadings, and Kaiser Normalisation to determine the number of factors to retain through the SPSS software.

The Pearson’s correlation matrix was used to evaluate the relationship between the water quality variables. A correlation coefficient near −1 or +1 means the strongest negative or positive relationship between two variables, with its value close to 0 means no linear relationship between them.

To examine the suitability of the present dataset for the PCA, some Kaiser–Meyer–Olkin (KMO) and Bartlett’s tests of Sphericity were performed. The KMO is a measure of sampling adequacy that represents the proportion of variance caused by underlying Principal Components. A higher value (between 0.5 and 1) generally indicates that the data set may be used for the PCA, which is the case in this data set: KMO=0.68. On the other hand, Bartlett’s test of Sphericity examines whether the correlation matrix is an identity matrix, in which case all variables become related, making the PCA model inappropriate and an unsuitable statistical tool for advanced data analysis. The Null Hypothesis of Bartlett’s test assumes that the correlation matrix is an identity matrix (i.e. there is no scope for dimensionality reduction). In the current case, the significance level equal to 0.000 (less than 0.05) rejects Null Hypothesis and shows significant relationships among the parameters.

The results of the PCA are reported in Tables S6, S7, S8, S9, S10, S11, S12, S13 and in Fig. S13. In this study, the two principal components that have eigenvalues > 1, and are included in the first set before a pronounced change of slope in the screen plot graphs (Fig. S13), were retained. The variables with eigenvalues lower than 1 were removed due to their low significance. The two components explain almost 56% of the total variance in the whole dataset (Table S12) and thus provide a general view on the temporal and spatial variations of water quality. If one considers seasonal variation, the situation is very similar with 54%, 52%, 55%, and 54% in winter, spring, summer, and autumn, respectively (Table S13). Only in autumn, the third component also appears with an eigenvalue slightly greater than 1. The first factor (PC1), accounting for 35% of the total variance, shows high positive loadings of *E. coli* and TP, and moderate positive loadings of NH_4_-N and BOD_5_. The *E. coli* parameter remains constantly high for the whole year, indicating a strong bacteriological component in the water deriving from untreated wastewater, animal husbandry, grazing areas, and the application of manure. TP decreases in spring due to less contributions of domestic and industrial discharges and of agricultural land fertilisers. NH_4_-N increases further in summer due to the negative impact of urban wastewater, indicating a high eutrophic risk of the river in this season. Finally, BOD_5_ shows lower values in autumn thanks to high flow regimes, which accelerate the purification processes. *E. coli* is positively correlated with NH_4_-N (r=0.380), TP (r=0.404) and BOD_5_ (r=0.322), confirming that they have a common origin and that thus only *E. coli* can be chosen as the representative parameter of PC1. The second factor (PC2) explains 56% of the total variance and has a positive high loading on DO and positive moderate loading on NO_3_-N. While oxygen remains high all year round and is slightly correlated with BOD_5_ (r=0.210), the NO_3_-N levels are quite low if one considers the seasonal trend.

**Table S6** p-values of water quality parameters for all seasons

|  | *E. coli* | NH_4_-N | NO_3_-N | TP | BOD_5_ | DO |
| --- | --- | --- | --- | --- | --- | --- |
| *E. coli* |  | 4.329‧10^-21^ | 2.990‧10^-2^ | 7.082‧10^-24^ | 2.384‧10^-15^ | 1.257‧10^-1^ |
| NH_4_-N | 4.329‧10^-21^ |  | 1.704‧10^-6^ | 2.362‧10^-6^ | 4.879‧10^-18^ | 2.290‧10^-6^ |
| NO_3_-N | 2.990‧10^-2^ | 1.704‧10^-6^ |  | 4.833‧10^-1^ | 6.181‧10^-7^ | 3.414‧10^-6^ |
| TP | 7.082‧10^-24^ | 2.362‧10^-6^ | 4.833‧10^-1^ |  | 3.702‧10^-17^ | 8.848‧10^-2^ |
| BOD_5_ | 2.384‧10^-15^ | 4.879‧10^-18^ | 6.181‧10^-7^ | 3.702‧10^-17^ |  | 2.364‧10^-7^ |
| DO | 1.257‧10^-1^ | 2.290‧10^-6^ | 3.414‧10^-6^ | 8.848‧10^-2^ | 2.364‧10^-7^ |  |

**Table S7** p-values of water quality parameters in: (a) winter, (b) spring, (c) summer, and (d) autumn





**Table S8** Correlation matrix of water quality parameters for all seasons

|  | *E. coli* | NH_4_-N | NO_3_-N | TP | BOD_5_ | DO |
| --- | --- | --- | --- | --- | --- | --- |
| *E. coli* | 1.000 | 0.380 | 0.079 | 0.404 | 0.322 | 0.048 |
| NH_4_-N | 0.380 | 1.000 | 0.194 | 0.192 | 0.351 | 0.192 |
| NO_3_-N | 0.079 | 0.194 | 1.000 | -0.002 | 0.203 | 0.188 |
| TP | 0.404 | 0.192 | -0.002 | 1.000 | 0.342 | -0.057 |
| BOD_5_ | 0.322 | 0.351 | 0.203 | 0.342 | 1.000 | 0.210 |
| DO | 0.048 | 0.192 | 0.188 | -0.057 | 0.210 | 1.000 |

**Table S9** Correlation matrix of water quality parameters in: (a) winter, (b) spring, (c) summer, and (d) autumn

**

**

**Table S10** Rotated factor loadings for water quality data in all seasons

|  | PCA1 | PCA2 |
| --- | --- | --- |
| *E. coli* | 0.781 | 0.022 |
| NH_4_-N | 0.565 | 0.428 |
| NO_3_-N | 0.061 | 0.684 |
| TP | 0.774 | -0.230 |
| BOD_5_ | 0.634 | 0.375 |
| DO | -0.016 | 0.752 |

**Table S11** Rotated factor loadings for water quality data in: (a) winter, (b) spring, (c) summer, and (d) autumn





**Table S12** Eigenvalues of water quality data set in all seasons

|  | Eigenvalues | Variance  (%) | Cumulative  Variance (%) |
| --- | --- | --- | --- |
| 1 | 2.108 | 35.127 | 35.127 |
| 2 | 1.237 | 20.617 | 55.744 |
| 3 | 0.813 | 13.557 | 69.301 |
| 4 | 0.731 | 12.189 | 81.490 |
| 5 | 0.607 | 10.115 | 91.605 |
| 6 | 0.504 | 8.395 | 100.000 |

**Table S13** Eigenvalues of water quality data set in: (a) winter, (b) spring, (c) summer, and (d) autumn

**

**

**
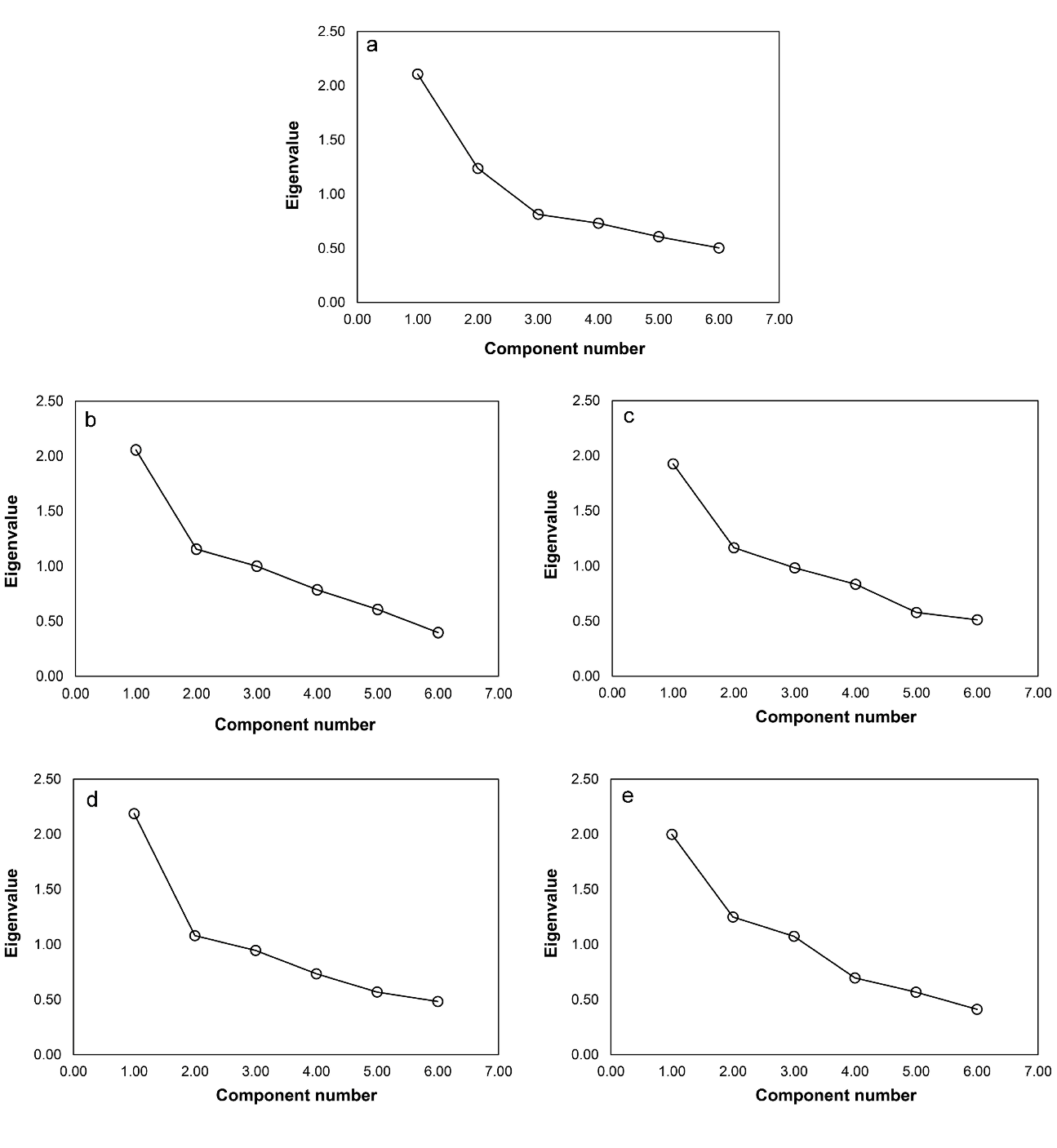
**

**Fig. S13** Screen plot of the eigenvalues in: (a) all seasons; (b) winter; (c) spring; (d) summer; and (e) autumn
